# Simultaneous Measurements of Auto-Immune and Infectious Disease Specific Antibodies Using a High Throughput Multiplexing Tool

**DOI:** 10.1371/journal.pone.0042681

**Published:** 2012-08-30

**Authors:** Atul Asati, Olga Kachurina, Anatoly Kachurin

**Affiliations:** Sanofi Pasteur, VaxDesign Campus, Orlando, Florida, United States of America; Lile 2 University, France

## Abstract

Considering importance of ganglioside antibodies as biomarkers in various immune-mediated neuropathies and neurological disorders, we developed a high throughput multiplexing tool for the assessment of gangliosides-specific antibodies based on Biolpex/Luminex platform. In this report, we demonstrate that the ganglioside high throughput multiplexing tool is robust, highly specific and demonstrating ∼100-fold higher concentration sensitivity for IgG detection than ELISA. In addition to the ganglioside-coated array, the high throughput multiplexing tool contains beads coated with influenza hemagglutinins derived from H1N1 A/Brisbane/59/07 and H1N1 A/California/07/09 strains. Influenza beads provided an added advantage of simultaneous detection of ganglioside- and influenza-specific antibodies, a capacity important for the assay of both infectious antigen-specific and autoimmune antibodies following vaccination or disease. Taken together, these results support the potential adoption of the ganglioside high throughput multiplexing tool for measuring ganglioside antibodies in various neuropathic and neurological disorders.

## Introduction

Monitoring antibodies against neuronal ganglioside antigens is necessary for the diagnosis and therapy of various immune disorders. Ganglioside-specific antibodies are known to participate in various immune mediated neuropathies such as Guillain-Barre syndrome (GBS), multifocal motor neuropathy (MMN), Miller Fisher syndrome (MFS), acute and chronic form of inflammatory demyelinating polyradiculoneuropathy (AIDP; CIDP) [1]–[9]. Moreover, ganglioside antibodies were found to have a role in the pathogenesis of the Alzheimer disease, and are suggested as peripheral blood biomarkers for Alzhiemer disease progression [10]. Various forms of multiple sclerosis (MS) have shown an increased level of circulating ganglioside antibodies that can serve as potential markers of axonal damage in MS [11]. Also, there are evidences connecting ganglioside antibodies with epilepsy, Sydenham chorea, autoimmune CNS inflammation and celiac disease [12]–[17]. Very recently, an elevated levels of GM1-ganglioside antibodies have been recently reported in mice after immunization against many influenza strains (1976, 1991–1992 and 2004–2005 vaccines) [18], [19]. Although conventional ELISA has been widely used for the detection of ganglioside antibodies [20]–[22], it has certain limitations such as considerable assay time, limited concentration sensitivity and lack of the multiplexing capacity that allows simultaneous detection of ganglioside and infectious antigen specific antibodies in a single sample volume. Alaedini et al [23], [24] reported an elegant express method to assess the presence of antibodies specific to the whole pool of neuronal gangliosides. The assay is based on agglutination of latex beads coated with the extract of human gangliosides with the antibodies. While being robust and time-saving, the method of Alaedini et al detects ganglioside antibodies at concentration 100–1000 times larger than the ELISA assays [24], lacks multiplexing capacity and is not able to discriminate antibodies specific to various gangliosides [23], [24].

Gangliosides are known as very labile compounds which make development of immunoassays complicated and may lead to false positive results [25]. Consequently, we reasoned that a more robust, specific, sensitive and multiplexing detection tool would be desirable for measuring ganglioside specific antibodies to help discern their roles in autoimmune disease and their usefulness as disease biomarkers. Considering a possible alternative between using multiplexing microarray ELISA-like technique and bead array BioPlex/Luminex platform, we decided in favor of the latter, due to the above mentioned instability of gangliosides [25]. We hypothesized that a reliable multiplexing system using Bioplex/Luminex beads can be designed to detect the presence of various ganglioside- and infectious disease-specific antibodies in a single sample volume.

## Results

### Synthesis and characterization of ganglioside-conjugated beads

**Figure 1:**
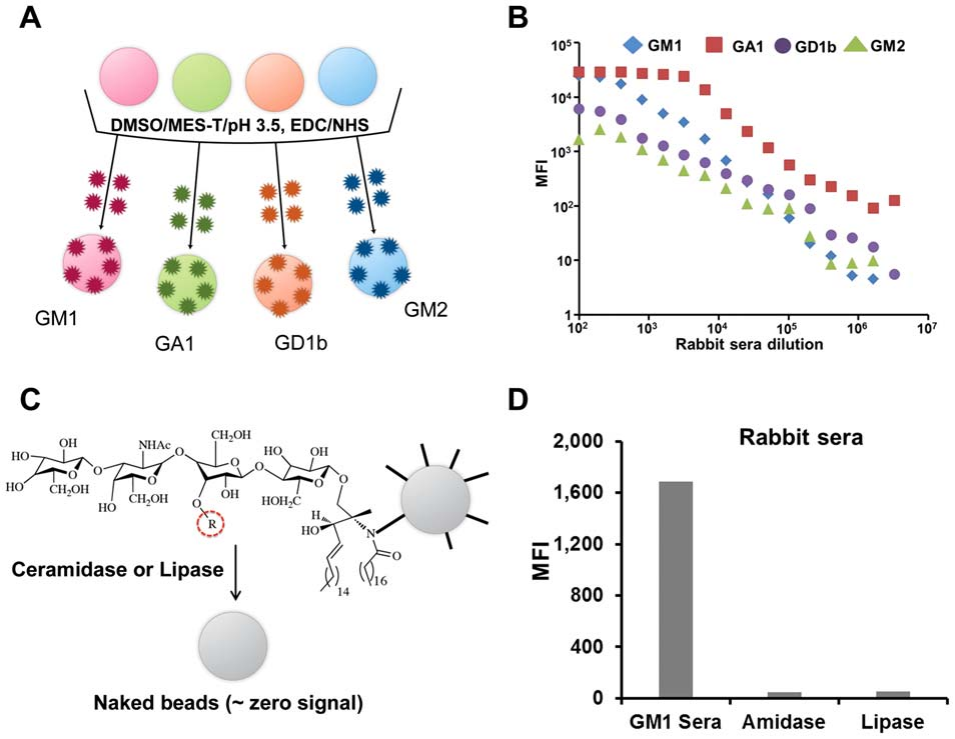
Synthesis and characterization of ganglioside bead array. A scheme of conjugation of various gangliosides on Bioplex beads via modified EDC/NHS chemistry (**a**). Dilution-dependent fluorescent response displayed by ganglioside conjugated beads testing various anti-ganglioside rabbit sera (**b**). Coupling of gangliosides to beads occurred via secondary amine next to ceramide moiety (**c**). Ceramidase and lipase treatment of beads negate the reporter fluorescent signal (**d**). The radical R circled in dashed red signifies the presence of sialic acid groups (one in GK1 and GM2, two in GD1b and none in GA1).

Ganglioside-conjugated bead arrays were fabricated using carbodiimide chemistry. A typical ganglioside molecule does not contain primary amine groups, which are typically used for conjugation with carboxyl groups, including those on the surface of Luminex beads which are used in the current study. However, we hypothesized that the conjugation of gangliosides could be achieved via the secondary amine groups adjacent to the ceramide moiety in ganglioside structure. Conjugation over another secondary amine group situated in the sialic acid residue was considered less feasible due to the possible steric hindrance. The gangliosides selected for coating the beads, GA1, GM1, GM2 and GD1b are known for clinical significance of the auto-antibodies towards these antigens in various neuropathic disorders [6]–[9]. The gangliosides were conjugated to the surface of carboxylated fluorescent Luminex beads, their code numbers 45, 27, 25 and 14 respectively, using a modified carbodiimide chemistry protocol (**Figure 1a** **; details of the protocol below**).

Manipulations with gangliosides in aqueous solutions create certain problems. Gangliosides can be easily dissolved in organic solvents such as DMSO (dimethyl sulfoxide), but develop micelles in watery buffers. Also, gangliosides easily lose their antigenic properties when stored in buffers at room temperature, which indicates conformational changes or chemical instability of the molecules. To avoid these problems, conjugation to the beads was carried out at 4°C in 1∶1 aqueous/organic mixture of MES-T buffer (0.05% v/v Tween 20 in 50 mM MES, pH 3.5) and DMSO. Upon completion of the conjugation reaction, the final ganglioside conjugated beads were washed in ice cold PBS and collected in PBS containing 2% w/v BSA (bovine serum albumin) and 0.1% w/v sodium azide. Successful conjugation of gangliosides to Luminex beads was confirmed by testing them with commercially available rabbit anti-ganglioside sera. Ganglioside-conjugated beads were able to capture the anti-ganglioside antibodies and display a dilution dependent fluorescent response (**Figure 1b**). Despite considerable hydrophobicity of the whole ganglioside molecules, their epitopes consist of hydrophilic carbohydrates and sialic groups. In order to reconcile the hydrophilic and hydrophobic propensities of the ganglioside components and to maintain proper conformations of the molecules on the Luminex beads, the tested sera and biotinylated detecting antibodies were diluted in aqueous/organic mixture of PBS/DMSO (70%/30% v/v). The streptavidin-phycoerythrin fluorescent tag (SA-PE) dissolved in 1% BSA was applied at the end of the assay. Coupling of gangliosides to the beads via secondary amide bond was assessed by the bond hydrolysis driven by ceramidase and lipase enzymes which are able to hydrolyze both primary and secondary amide bonds (**Figure 1c**) [26]–[28]. As expected, Bioplex experiments showed lack of the reporter fluorescent signal after treatment of beads with ceramidase or lipase (**Figure 1d**). Successful coupling of the asialo-GM1 ganglioside to the beads further confirmed our hypothesis that conjugation took place via the secondary amine group adjacent to ceramide moiety, since asialo-GM1 ganglioside does not possess a sialic acid and the sialic secondary amine group. Another consideration in favor of the conjugation of gangliosides via ceramide-adjacent nitrogen is a sheer fact that the carbohydrate and sialic acid moieties serve as key ganglioside epitopes [29], [30]. If the conjugation would have occurred via nitrogen other than the one adjacent to ceramide moiety, the epitopes would not be readily available for ganglioside-specific antibodies.

Having the ganglioside bead arrays fabricated and tested with anti-ganglioside sera, we performed selection of the optimal secondary detecting antibody. For this purpose, a standard human anti-GM1 serum from Buhlmann ELISA kit (EK-GM1-GM, Buhlmann Laboratories AG, Switzerland) was used as the test sample. Various secondary antibodies from different vendors were applied such as goat anti-human IgG:biotin (Southern Biotech), goat anti-human (IgG+IgM)(H+L):biotin, donkey anti-human IgG(H+L)Fab_2_:biotin and goat anti-human IgG(Fc):biotin (all three from Jackson Immunoresearch). We found that donkey anti-human IgG(H+L) Fab_2_:biotin provided a considerably higher reporter signal compared to the other secondary antibodies (**Figure S1**), and this antibody was used in the subsequent Bioplex measurements of ganglioside-specific IgG.

### Epitope specificity and cross reactivity determination of ganglioside high throughput multiplexing tool

**Figure 2:**
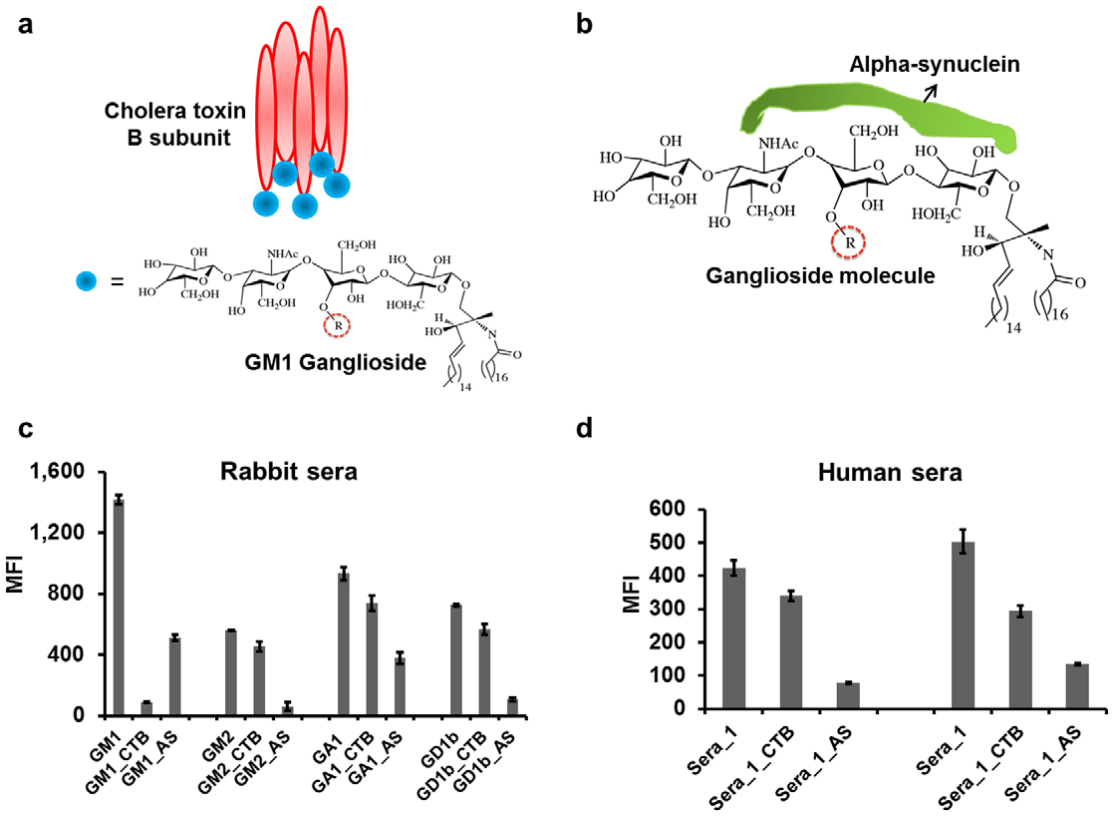
Evaluating bead epitope specificity using CTB and AS epitope blockers. CTB has ability to bind 5 GM1 molecules (**a**) compared to alpha-synuclein that can bind only one GM1 molecule (**b**). CTB showed ∼90% bead blocking with anti-GM1 rabbit serum compared to ∼15% with other anti-ganglioside rabbit sera while synuclein exhibits ∼60–90% blocking with all anti-ganglioside sera (**c**). Screening human sera, 20%–40% blocking was seen with CTB compared to 70%–80% using AS (**d**). The radical R circled in dashed red signifies the presence of sialic acid groups (one in GK1 and GM2, two in GD1b and none in GA1).

It is well known that the cholera toxin beta subunit (CTB) conjugates specifically and strongly with the carbohydrate/sialic acid epitope of the GM1-ganglioside but has considerably lower affinity towards other gangliosides [31], [32]. Another potential blocker, alpha-synuclein (AS) binds specifically to carbohydrate and sialic acid residues of GM1-ganglioside [33] (**Figure 2a and 2b**). Although the binding capacity of alpha-synuclein to other gangliosides is not well characterized. We carried out the epitope specificity tests using both CTB and AS as ganglioside epitope blockers. The ganglioside-conjugated beads were individually pre-incubated with each blocker, and after that rabbit anti-ganglioside sera were added. As expected, CTB was able to block almost 100% of the signal from the GM1 coated beads, and showed much lower effect on beads coated with other gangliosides (**Figure 2c**). Alpha-synuclein showed affinity to all ganglioside-conjugated beads blocking from 60% to 90% of fluorescent signal (**Figure 2c**). However, in the measurements of human sera, only 20% to 40% blocking with CTB was observed while AS showed 70% to 80% blocking (**Figure 2d**). This result assumes that human sera may contain GM1-reacting antibodies specific to the epitopes other than the known carbohydrate/sialic acid epitope, probably including a certain part of the ceramide moiety. Similarly, effects of blockers were assessed by performing ganglioside-specific ELISA for GM1, GM2, GA1 and GD1b ganglioside (Figure S2). The ELISA assay demonstrated blocking effect of CTB with GM1 ganglioside only, and no blocking effect of AS with each of the four gangliosides. Additionally, we tested the effect of blockers using commercially available Buhlmann anti-human GM1 sera standard. Buhlmann ELISA has been designed to quantitatively determine IgG/IgM isotypes of autoantibodies directed against GM1 in human serum applying individual IgG- and IgM-specific conjugates. Interestingly, using internal Buhlmann anti-GM1 sera standard in the ELISA experiment, we did not see any blocking effect of CTB or AS (Figure S3), Blockers assessment with other ganglioside-specific human sera standards such as anti-GA1, anti-GM2 or anti-GD1b were not done in this study due to their commercial unavailability. We assume that lack of blocking capacity in the ELISA experiments could be explained by a different orientation of gangliosides bound on the ELISA plates compared to the gangliosides conjugated to the bead array, where they are oriented in a pendant-like projection readily available for binding with the blockers, while in the ELISA plate this favorable orientation may not be possible. These results demonstrate the unique ability of multiplexing tool over ELISA to demonstrate epitope specificity of the ganglioside-reacting antibodies using epitope blockers such as CTB and AS.

**Figure 3:**
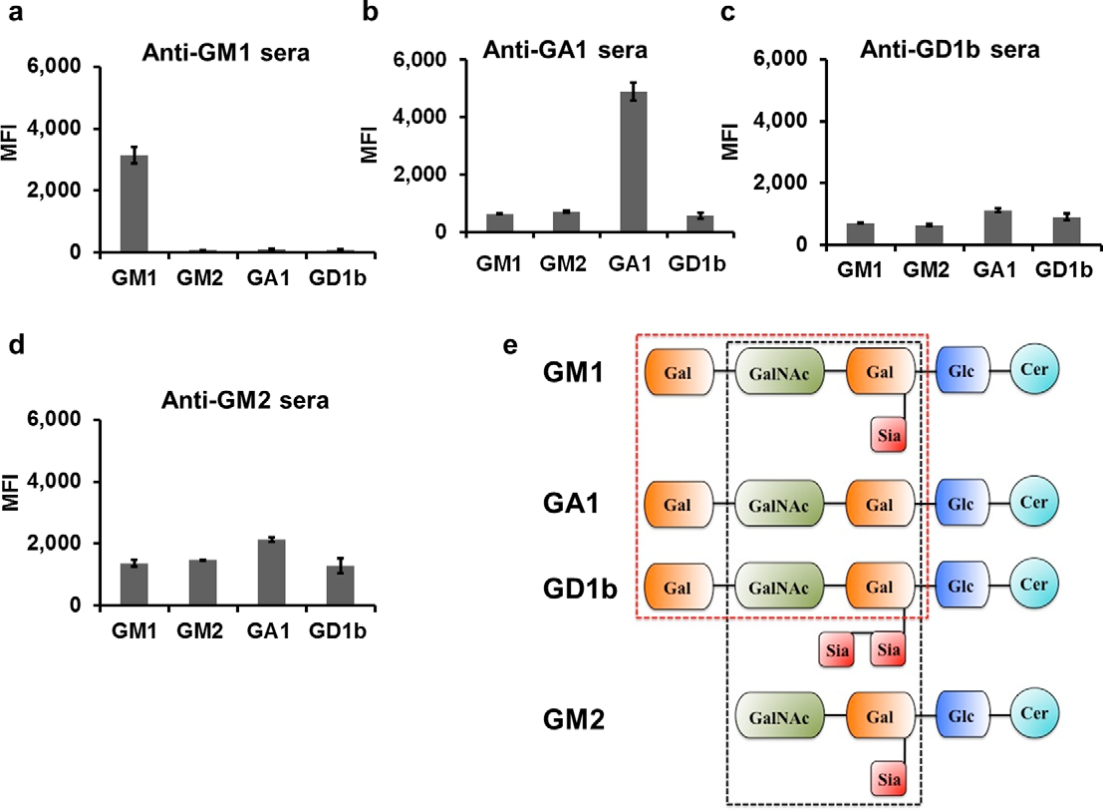
Cross-reactivity of ganglioside antibodies. GM1- and GA1-conjugated beads showed considerable cross reactivity with anti-GD1b sera since these gangliosides have common terminal sugar sequence **(panels b, c; dotted red square in panel e)**. GM1 beads displayed cross-reactivity with anti-GM2 serum due to the shared epitope groups in the inner part of the GM1- and GM2-gangliosides **(panel d; dotted black square in panel e)**. Cross-reactivity was estimated comparing the reporter signal from the beads coated with the ganglioside related to the tested serum and the signals from the beads coated with other gangliosides.

In order to further characterize specificity of the ganglioside HTM tool, cross reactivity of ganglioside-conjugated beads were tested using commercially available anti-ganglioside rabbit sera. A typical ganglioside molecule contains a carbohydrate sequence with a varying number of sialic acid residues attached to the carbohydrate backbone [31],[33]. The structural similarities among various gangliosides may result in cross reactivity of ganglioside antibodies to the beads coated with different gangliosides which may affect the assay results. ELISA and TLC (thin layer chromatography) have been used by others to assess the cross reactivity of ganglioside specific antibodies [34], [35], but the new ganglioside HTM tool needed to be characterized independently. A significant cross reactivity for anti-GM2 and anti-GD1b sera compared to anti-GM1 and anti-asialo-GM1 sera was found (**Figure 3a, 3b, 3c, 3d**). GM1-, asialo-GM1- and GD1b-gangliosides have common terminal sugar sequence (**Figure 3e** **, dotted red square**), hence beads coated with GM1-ganglioside and asialo-GM1 ganglioside exhibit a significant cross reactivity to the anti-GD1b sera. In the same time, general prediction of the sera cross reactivity can be complicated by its dependence on the particular epitope spectra of the antibodies included in the anti-sera. For example, cross reactive antigen-antisera relations for GM1 and GM2 gangliosides were found asymmetrical; for example anti-GM1 serum did not react with GM2 bead while anti-GM2 serum did react with GM1 bead. Hypothetically, the terminal galactose of the GM1 ganglioside was a critical part of the major reactivity epitope for the anti-GM1; therefore lack of the terminal galactose in the GM2 banned interaction of anti-GM1 serum with GM2 bead (**Figure 3e**). On the other hand, the major epitope of the anti-GM2 can include Gal-NAc-Gal-Sialic Acid construct common for all four gangliosides used in the array. The cross reactivity of GM1-beads towards anti-asialo-GM1 serum was very insignificant, which reflected the importance of sialic moiety in shaping up the GM1 epitopes. Also, lack of cross reactivity of GD1b-ganglioside beads towards anti-GM1 and anti-asialo-GM1 sera can be attributed to the extra sialic acid in the GD1b structure, which may prevent binding of anti-GM1 and anti-asialo-GM1 antibodies due to the steric effect.

### Comparing concentration sensitivity of the multiplexing tool and ELISA

**Figure 4:**
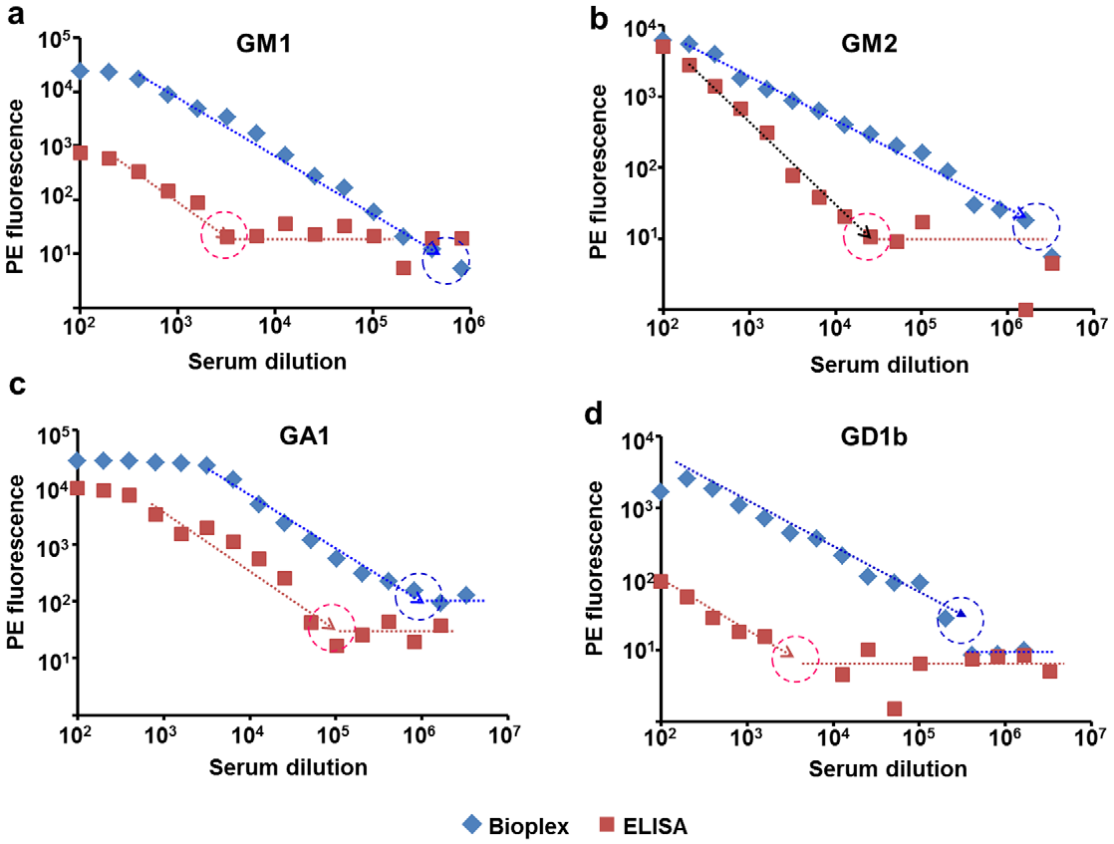
Concentration sensitivity to ganglioside-specific sera of BioPlex bead array, compared to ELISA. Ganglioside-conjugated Luminex bead exhibited approximately 100 times better sensitivity to the sera specific to GM1, GM2 and GG1b gangliosides (a, b, d), and about 10 times better sensitivity to the serum specific to GA1 (c). Both BioPlex and ELISA assays used the same detecting antibody, anti-rabbit IgG(H+L)Fab2:biotin at 2 ug/ml, and SA-PE fluorescent tag at 4 ug/ml. Dashed circles show approximate sensitivity thresholds for BioPlex (blue) and ELISA (red).

As stated earlier, ELISA is widely used for the detection of ganglioside antibodies, but concentration sensitivity of ELISA assay is limited. We assumed that ganglioside-conjugated beads may exhibit better concentration sensitivity than ELISA due to the more appropriate orientation and better exposure of the ganglioside epitopes. We compared the concentration sensitivity of ganglioside-conjugated Luminex beads and the traditionally used ganglioside-specific ELISA for all four mentioned gangliosides GM1, GM2, GA1 and GD1b. Experiments were carried out with the commercially available standard rabbit anti-ganglioside serum as a tested sample, using two-fold serial dilution from 1∶100. For both the Bioplex and ELISA experiments, biotinylated donkey anti-rabbit IgG(H+L) Fab2:biotin was used for measuring IgG fraction, with SA-PE as a fluorescent tag. The concentration sensitivity of the ganglioside-conjugated beads in the BioPlex experiment was found 10–100 times better than the ELISA assay for IgG detection, (Figure 4a, 4b, 4c, 4d). Similar comparison of the concentration sensitivity was performed using internal anti-GM1 standard. In this case, the ELISA assay used the original Buhlmann protocol and colorimetric registration. The concentration sensitivity of the ganglioside-conjugated beads in this test was found ∼50 times better than in the ELISA assay for IgG and ∼100 times better for IgM (Figure S3).

### Demonstration of multiplexing capacity for infectious-disease specific antibodies

**Figure 5:**
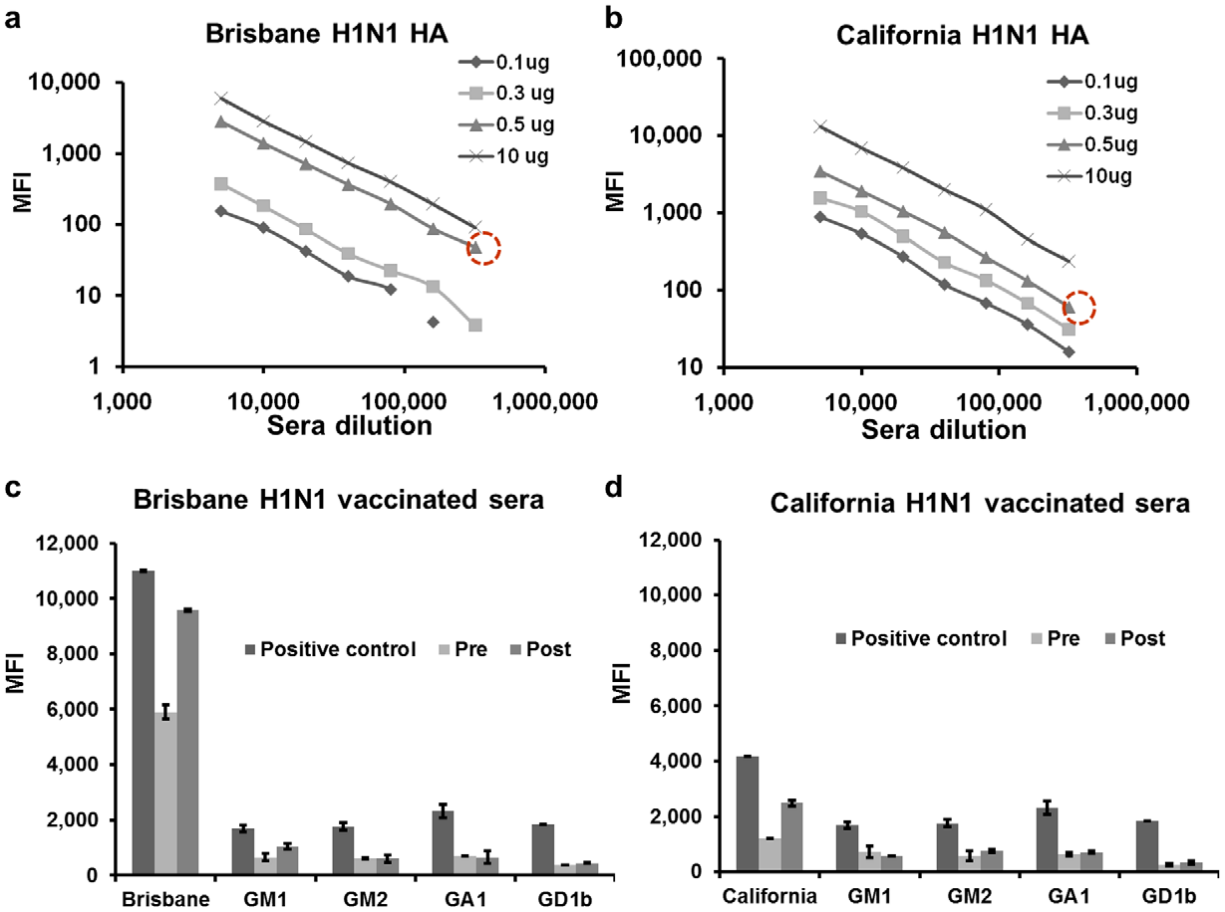
Testing multiplexing capacity of combined ganglioside-influenza bead array. Luminex beads coated with various payloads of Brisbane H1N1 and California H1N1 hemagglutinins were tested, and beads with the payload 0.5 µg (encircled in red) was selected for the combined ganglioside-influenza bead array (a, b). As a proof of principle, ganglioside-influenza bead array was tested with sera from two donors vaccinated against seasonal influenza in 2009 (c) and against pandemic California H1N1 in 2010 (d). Increase of influenza-specific antibodies post vaccination is apparent. More details in the text. (Note: Errors were too small to include in the panels a and b). Anti-Brisbane H1N1 and anti-California H1N1 sheep sera (NIBSC, UK) were used at dilution 1∶ 50000 as a positive control for detecting influenza-specific antibodies by hemagglutinin-coated beads. Anti-ganglioside rabbit sera (Matreya LLC) were used as positive controls for detecting ganglioside-specific antibodies at the following dilutions: anti-GM1 - 1∶6400, anti-GM2 - 1∶800, anti-GA1 – 1∶25000, anti-GD1B – 1∶400.

In our previous experiments with influenza bead arrays, the concentration sensitivity of influenza coated beads was found very high, and the dilutions normally used in measuring influenza-positive sera were from 1∶50000 to 1∶500000 (data not shown). Using such dilutions was not appropriate for simultaneous detection of ganglioside-specific autoantibodies in human sera, since the auto-antibodies are scarce compared to the antibodies of the influenza vaccination response. In order to allow simultaneous measurements of influenza- and ganglioside-specific antibodies with the same bead array, the sensitivity of the influenza beads was intentionally reduced by decreasing the payload of influenza hemagglutinins on the beads. This approach allowed screening of sera at dilutions 1∶100–1∶2000 (**Figures 5a and 5b**). As a proof of the principle, human sera from donors immunized with vaccines containing Brisbane H1N1 and California H1N1 components were tested at dilution 1∶2000 using pentaplex bead array containing various ganglioside and influenza beads. As positive controls, anti-Brisbane H1N1 and anti-California H1N1 sheep sera were used for hemagglutinin-coated beads, and ganglioside-specific rabbit sera for ganglisoide-coated beads. As expected, the multiplex tool successfully screened both influenza and ganglioside antibodies in a single sample volume (Figure 5c and 5d). In addition to assessing the levels of ganglioside-specific antibodies, those multiplexing measurements showed a 2-fold increase in influenza-specific antibodies [36], [37] post vaccination, but no change in the ganglioside-specific antibodies. The experiment therefore demonstrated the capacity of the multiplex tool to assess both infectious disease and autoimmune responses in a single run and the fact that influenza immunization normally does not cause autoimmunity.

## Discussion

Concluding, we report development of a novel high throughput multiplexing tool based on the bead array technology, which provides simultaneous measurements of autoimmune antibodies specific to various neuronal gangliosides together with antibodies to an infectious disease (influenza). The bead array was fabricated using modified carbodiimide chemistry in an aqueous/organic media, where linking of the gangliosides occurred via the secondary amine group adjacent to the ceramide moiety. The ganglioside-influenza bead array exhibited excellent detection limits offering 10–100 times better concentration sensitivity to ganglioside-specific antibodies than the ELISA assay, including the golden standard, Buhlmann GM1 ELISA kit. Moreover, the array demonstrated excellent reactivity towards ganglioside blockers, CTB and AS, which proved epitope specificity of the detected antibodies. Combining ganglioside- and influenza-specific elements in one instrument provided an exclusive capability to measure immune response to infectious disease or vaccination and their possible autoimmune effects in a simultaneous assay. Therefore, potential application of such type of a multiplexing instrument can be expected in diagnosis and monitoring of the neuropathic or neurological disorders, and in investigating their possible relations with infectious diseases or vaccination.

## Materials and Methods

### Fabrication of beads coated with gangliosides

All gangliosides were purchased from American Research Products, INC. For bead array fabrication, fluorescent carboxylated non-magnetic beads (Luminex) were used, #45 for GM1 ganglioside, #27 for GM2 ganglioside, #25 for GA1 asialo ganglioside, #14 for GD1b diasialo ganglioside. First, 50 µl of bead suspension (from stock 1.25*10^7^ beads/ml) was diluted in 270 µl of 100 mM, pH 3.5 MES (2-(*N*-morpholino)ethanesulfonic acid) buffer containing 0.05% Tween-20 (MES-T, all components from Sigma) and activated using 100 µl of ethyl(dimethylaminopropyl) carbodiimide (EDC) (100 mg/ml) with immediate addition of 100 µl of N-hydroxy succinamide NHS (100 mg/ml) (both from Pierce Biotechnology) and the reaction was carried out for 20 minutes at room temperature.Afterwards, beads were washed with MES-T buffer using centrifugation at 600 G for 8 minutes on 3-µm Transwell inserts (Life Technologies) assembled on top of 15-ml centrifuge tubes. Washing procedure was repeated twice in order to remove excess of unreacted EDC and NHS. Afterwards, the beads were aspirated into a 1.5-ml Eppendrof tube using 150 µl of DMSO/MES-T (50%/50% mixture) and mixed with 30 µg of ganglioside, respective to the selected bead number. Reaction was allowed to go for 24 hours on a planetary shaker at 600 rpm and 4°C, and then quenched by adding 600 µl of 50 mM glycine buffer pH 7.3. The resultant mixture was incubated for 15 hours at 4°C and 600 rpm shaking. Upon completion, the final gangliosides-conjugated beads were washed in cold PBS through centrifugation at 600 G for 8 minutes using 3-µm Transwell inserts assembled on tops of 15-ml centrifuge tubes and collected in PBS containing 2% Grade V BSA (bovine serum albumin) and 0.1% sodium azide (Sigma). Finally, 10% v/v of glycerol was added, and the bead suspension was stored in liquid nitrogen. The bead number was calculated using a hemocytometer.

### Testing of a standard anti-gangliosides rabbit serum

Standard rabbit sera against the gangliosides GM1, GM2, GA1 and GD1b were obtained from Matreya LLC. Each serum was serially diluted two-fold starting from 1∶100 to 1∶3276800. Sera dilution was made in 70/30 mixture of PBS/DMSO. 100-µl portions of the diluted anti-ganglioside rabbit sera were incubated in duplicates with corresponding ganglioside-conjugated beads in a 96-well Bioplex filter plates (Bio-Rad) at 4°C for 150 minutes at 600 rpm shaking. Afterwards, the wells were washed twice with 85 µl of PBST/NaN_3_ for 2 minutes on a planetary shaker at 600 rpm at room temperature filtering the plates on a vacuum manifold after each wash. Afterwards, 85 µl (1 µg/ml in 70/30 PBS/DMSO) of biotinylated anti-rabbit IgG(H+L)Fab_2_:biotin (Jackson Immunoresearch) was applied as a detecting antibody, and the plate was incubated at 4°C for 40 minutes at 600 rpm. Again, the wells were washed twice with PBST/NaN_3_ as described above, and 85 µl of 4 µg/ml of streptavidin-phycoerythrin (SA-PE) fluorescent conjugate (Leinco Technologies, Inc.) was introduced into each well. After incubation for 40 minutes at 4°C, the plate was washed twice with PBST/NaN_3_ as described above, and once with PBS/NaN_3_ containing no detergent. Then 85 µl of PBS/NaN_3_ was added to each well, and the plate was shaken for 5 minutes at 1300 rpm at room temperature to avoid settling of the beads. Finally, another 55 µl of PBS/NaN_3_ was added to each well, and the plate was shaken at 600 rpm for one minute. After that, the plate was read on Bioplex-100 bead array reader (Bio-Rad). As a negative (mock-) control, 100 µl mixture of PBS/DMSO was used. Similar protocols were used for measuring or detecting ganglioside antibodies in all assays, including studying human donor sera. All the experiments were carried out in duplicates.

### BioPlex experiment after ceramidase/lipase treatment

Individual beads were incubated in 10 µg/ml ceramidase or lipase (Sigma Aldrich) at 4°C overnight at 600 rpm shaking. Then the treated beads were incubated in duplicates with 100 µl of anti-ganglioside sera at 4°C for 150 minutes at 600 rpm. Remaining procedure was followed as described in the above section.

### Selection of optimal detecting antibodies

Four different types of detecting antibodies were tested using gangliosides-conjugated beads and human anti-GM1 gangliosides serum from Buhlmann GM1 ELISA kit. For IgG, goat anti-human IgG:biotin (Southern Biotechnology), goat anti-human IgG(H+L)Fab_2_:biotin, goat anti-human (IgG+IgM)(H+L)Fab_2_:biotin and donkey anti-human IgG(H+L)Fab_2_:biotin (all three from Jackson Immunoresearch) were screened. For IgM, goat anti-human IgM:biotin (Southern Biotechnology), Goat anti-human IgM(H+L)Fab_2_:biotin, goat anti-human (IgG+IgM)(H+L)Fab_2_:biotin and donkey anti-human IgM(H+L)Fab_2_:biotin (all three from Jackson Immunoresearch). Bioplex experiment was set as described above with only difference of using human anti-GM1 ganglioside sera instead of the rabbit sera, and applying human specific rather than rabbit specific detecting antibodies. Sera and detecting antibody were diluted in 70%/30% PBS/DMSO mixture. All antibodies were tested in duplicates.

### Evaluation of epitope specificity employing CTB and AS ganglioside blockers – Bioplex and ELISA assays using the same components

To test epitope specificity in the BioPlex experiment, the standard Bioplex protocol described above was followed, except that the individual ganglioside conjugated beads were pre-incubated with 50 µl of CTB (2 µg/ml) (Sigma Aldrich) or 50 µl of AS (15 µg/ml) (Sigma Aldrich) in duplicates in a 96-well plate at 4°C for 1 hour at 500 rpm shaking. Then 50 µl of anti-rabbit ganglioside sera were introduced into respective wells (1∶6400 of anti-GM1, 1∶50000 of anti-GA1, 1∶1600 of anti-GM2 and 1∶800 of anti-GD1b sera). After performing the whole procedure, the plate was read in the Bioplex reader. A similar experiment was carried out employing human sera at dilution of 1∶2000, but donkey anti-human IgG(H+L)Fab_2_:biotin (Jackson Immunoresearch) was used as a detecting antibody instead of anti-rabbit IgG(H+L)Fab_2_:biotin. All sera dilutions and detecting antibody solutions were made in 70%/30% PBS/DMSO mixture. Similarly, ELISA assays were carried out by coating the Immulon 4HBX strips with 100 µl per well of ganglioside solutions at 5 µg/ml in duplicates, in 70/30 mixture of PBS/DMSO overnight. Next day, the plates were blocked with 200 µl/well of 2% BSA for 2 hours. Then plates were incubated with 50 µl of CTB (2 µg/ml) (Sigma Aldrich) or 50 µl of AS (15 µg/ml) (Sigma Aldrich) at 4°C for 1 hour at 500 rpm shaking. Then plates were washed twice with PBST and respective anti-rabbit ganglioside specific sera were introduced. After incubation for 2 hours, the plates were washed twice with PBST and anti-rabbit IgG(H+L)Fab2:biotin detecting antibody was added at 2 µg/ml, 85 µl/well, and incubated for 1 hour. Next, the plates were washed with PBST thrice and incubated with 85 µl/well of 4 µg/ml of SA-PE for 1 hour. Finally, the plates were washed with PBST four times and read on BioTek plate reader using phycoerythrin filter setting. All the experiments were carried out in duplicates.

### Buhlmann GM1 ELISA – a golden standard assay

Buhlmann ELISA kit for GM1-specific antibodies (IgG and IgM) was used as a golden standard assay in this study. The kit (EK-GM1-GM, Buhlmann Laboratories AG, Switzerland) contains a microtiter plate coated with GM1 antigen (the formulation of which is not specified in the product manual), and blocked by the manufacturer. The kit also contains calibrator and control (standard) solutions of GM1-specific sera, detecting antibodies tagged with horseradish peroxidase (HRP), TMB peroxidase substrate, a stop solution and washing and dilution buffers. The ELISA is based on a regular colorimetric technique using peroxidase driven oxidation of the TMB substrate into a blue chromophore and turning it into a yellow chromophore using an acidic stop solution. The optical density of the yellow chromophore is read in the plate reader at 450 nm. The whole procedure of applying calibrator, control and tested samples, washings, application of the detecting antibodies, reading takes ∼4.5 hours [38].

Buhlmann Labs also supply separately the GM1-specific human sera standard, which makes it a convenient sample for comparative sensitivity tests.

### Evaluation of epitope specificity using CTB and AS blockers in Buhlman GM1 ELISA kit

ELISA strip coated with GM1-ganglioside as provided in the kit was pre-incubated with 50 µl of CTB (2 µg/ml) and 50 µl of AS (15 µg/ml). The rest of the procedure was performed following the protocol supplied in Buhlmann ELISA kit. All the measurements were carried out in duplicates.

### Evaluation of reactivity

For cross reactivity evaluation, each type of ganglioside-conjugated beads was incubated with 100 µl of particular dilution of the corresponding anti-ganglioside sera (1∶6400 of anti-GM1, 1∶50000 of anti-GA1, 1∶1600 of anti-GM2 and 1∶800 of anti-GD1b sera) in duplicates in a 96-well plate at 4°C for 150 minutes at 600 rpm. The dilutions were chosen to obtain comparable reporter signals for different sera, based on the previous sera titrations. The sera and anti-rabbit IgG(H+L)Fab_2_:biotin detecting antibody were diluted in 70%/30% PBS/DMSO mixture. Bioplex measurement protocol was used as described in the earlier sections. Cross-reactivity was estimated by comparing the reporter signal from the beads coated with the ganglioside related to the tested serum and the signals from the beads coated with other gangliosides.

### Comparing concentration sensitivity of ganglioside-conjugated bead array and ELISA

Concentration sensitivity measurements were done using either ganglioside-specific rabbit sera, or Buhlmann GM1 sera standard, in parallel for both BioPlex and ELISA measurements. Two-fold serial dilution starting from 1∶100 was used. Sera samples were diluted in 70%/30% PBS/DMSO mixture. Ganglioside conjugated beads were incubated with 100 µl of serially diluted sera for 2.5 hours. After bead washing, 85 µl of goat anti-rabbit IgG(H+L)Fab_2_:biotin or donkey anti-human IgG(H+L)Fab_2_:biotin detecting antibody were applied as a detecting antibody, and SA-PE used as a fluorescent tag. BioPlex measurement procedure was used as described in the earlier sections.

In-house ELISA was carried out by coating the Immulon 4HBX strips with 100 µl of 5 µg/ml ganglioside solution in 70/30 mixture of PBS/DMSO per well in duplicates, overnight. Next day, the plates were blocked with 200 µl/well of 2% BSA for 2 hours. Then the plates were washed twice with PBST and respective rabbit anti-ganglioside sera were introduced, serially diluted starting with dilution 1∶100. After incubation for 2 hours, the plates were washed with PBST twice, and anti-rabbit IgG(H+L)Fab2:biotin antibody was added at 2 µg/ml, 85 µl/well, and the plates incubated for 1 hour. Next, the plates were washed with PBST thrice and incubated for 1 hour with 85 µl/well of of SA-PE at 4 µg/ml. Finally, the plates were washed with PBST four times and read on BioTek plate reader using phycoerythrin filter setting. All the experiments were carried out in duplicates.

Buhlmann ELISA using the internal GM1 sera standard as a sample was carried out according to the Buhlmann kit protocol. The GM1 specific sera standard was applied serially diluted, starting with dilution 1∶100., in duplicates.

### Fabrication of beads coated with influenza hemagglutinin

Fluorescent carboxylated non-magnetic beads (Luminex) were used, #42 for California H1 recombinant hemagglutinin, and #12 for Brisbane H1 recombinant hemagglutinin (both from Protein Sciences). First, 50 µl of bead suspension (from stock 1.25*10^7^ beads/ml) was diluted in 270 µl of 100 mM pH 3.5 MES-T buffer and activated using 40 µl of ethyl(dimethylaminopropyl) carbodiimide (EDC; 100 mg/ml, Pierce Biotechnology) and 40 µl N-hydroxy succinamide (NHS, 100 mg/ml, Pierce Biotechnology) for 20 minutes at room temperature. Afterwards, beads were washed with MES-T buffer through centrifugation at 600 G for 8 minutes, using 3-µm Transwell inserts as described above. Washing procedure was repeated twice in order to remove excess of unreacted EDC or NHS. Then the beads were aspirated into a 1.5-ml Eppendrof tube using 150 µl of MES-T and an aliquot of 10 µg, 0.5 µg, 0.3 µg or 0.1 µg of hemagglutinin was admixed. The reaction was allowed to go for 24 hours at 4°C on a planetary shaker at 600 rpm and then quenched by adding 600 µl of 50 mM glycine buffer pH 7.3 (Sigma). The mixture was incubated for 15 hours at 4°C on a planetary shaker at 600 rpm. Upon completion of conjugation reaction, the final gangliosides-conjugated beads were washed in cold PBS through centrifugation at 600 G for 8 minutes using 3-µm Transwell inserts as described above, and collected in PBS containing 2% BSA (bovine serum albumin) and 0.1% sodium azide. Finally, 10% v/v of glycerol (Sigma) was added, and the bead suspensions were stored in liquid nitrogen. The bead number was calculated using a hemocytometer.

### Demonstration of multiplexing capacity of ganglioside and influenza bead array

Human pre- and post-vaccinated sera collected from donors vaccinated against seasonal influenza in 2009 and against pandemic California H1N1 virus in 2010 were tested at dilution 1∶2000 in the 70%/30% PBS/DMSO mixture. Prior to incubation with sera samples, the beads were combined in a pentaplex array containing GM1-, GM2-, GA1-, GD1b-conjugated beads, and Brisbane H1 HA coated beads, or California H1 HA coated beads. Human sera samples were incubated with bead arrays for 150 minutes at 4°C at 600 rpm. After that, the plate was washed twice with PBST/NaN_3_ and then 85 µl (2 µg/ml in 70/30 PBS/DMSO mixture) of anti-human donkey IgG(H+L) Fab_2_:biotin was applied as a detecting antibody. The remaining washing procedure, application of SA-PE and reading on Bioplex were similar to the BioPlex protocol described in the earlier sections. Anti-Brisbane H1N1 and anti-California H1N1 sheep sera (NIBSC, UK) were used at dilution 1∶50000 as a positive control for detecting influenza-specific antibodies by hemagglutinin-coated beads. Anti-ganglioside rabbit sera (Matreya LLC) were used as positive controls for detecting ganglioside-specific antibodies at the following dilutions: anti-GM1 - 1∶6400, anti-GM2 - 1∶800, anti-GA1 – 1∶25000, anti-GD1B – 1∶400.

## Supporting Information

Figure S1 **Selection of optimal secondary detecting antibody.** Donkey IgG(H+L)Fab2:biotin from Jackson Immunoresearch was selected for detection of ganglioside-specific IgG and for most of the BioPlex experiments. MFI = Mean Fluorescence Intensity.
(TIF)

Figure S2 **Testing epitope specificity using CTB and AS blockers in ELISA assays.** GM1-specific sera standard was used as a sample in Buhlmann GM1 ELISA, and no blocking with either CTB or AS was observed (a). Effective blocking of GM1 by CTB was demonstrated in the in-house fluorescent ELISA, but no blocking effects of both CTB and AS were observed on the other gangliosides (b). Similar experiments using ganglioside Luminex bead array showed effective blocking of gangliosides with CTB and AS (Figure 2 in the main part).
(TIF)

Figure S3 **Concentration sensitivity to ganglioside-specific sera of BioPlex bead array, compared to Buhlmann ELISA.** GM1-specific sera standard from Buhlmann kit was used as a sample in both BioPlex and ELISA experiments. Buhlmann GM1 ELISA was set using the kit protocol. In order to enable side-by side-comparison with Bioplex fluorescent data, ELISA optical density data were multiplied by arbitrary factors of 104 (IgG measurements, panel a) or 103 (IgM measurements, panel b).
(TIF)
