# Supplementary figures and images for: Simultaneous Measurements of Auto-Immune and Infectious Disease Specific Antibodies Using a High Throughput Multiplexing Tool
Source: PLoS One. 2012 Aug 30;7(8):e42681. doi: 10.1371/journal.pone.0042681 (PMC3431397; doi:10.1371/journal.pone.0042681)

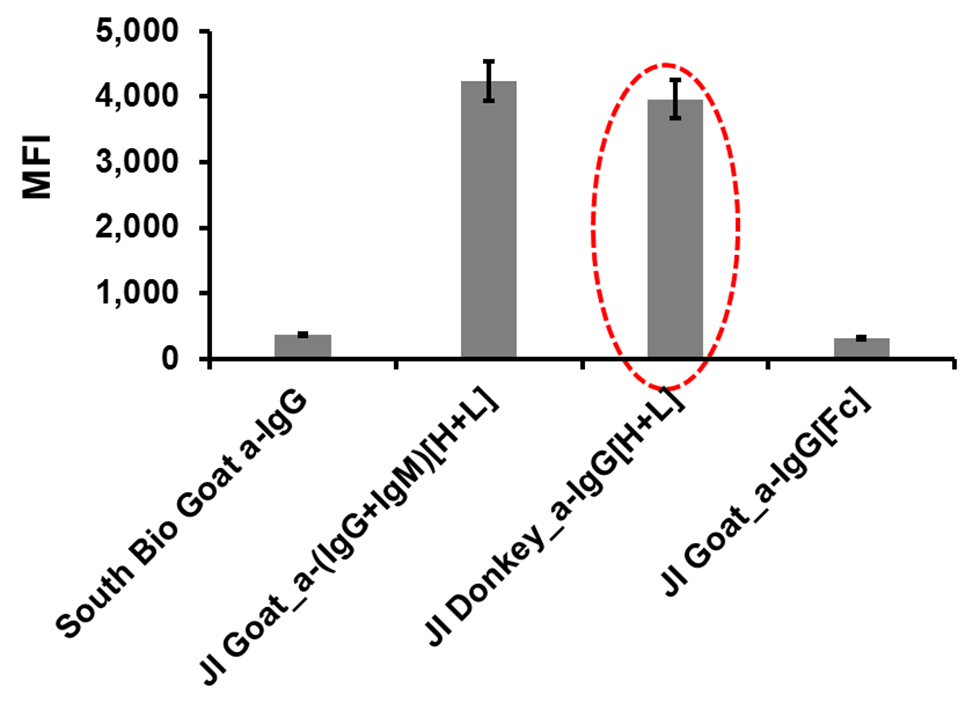

Supplement: Figure S1 — Selection of optimal secondary detecting antibody. Donkey IgG(H+L)Fab2:biotin from Jackson Immunoresearch was selected for detection of ganglioside-specific IgG and for most of the BioPlex experiments. MFI = Mean Fluorescence Intensity. (TIF) [file pone.0042681.s001.tif]

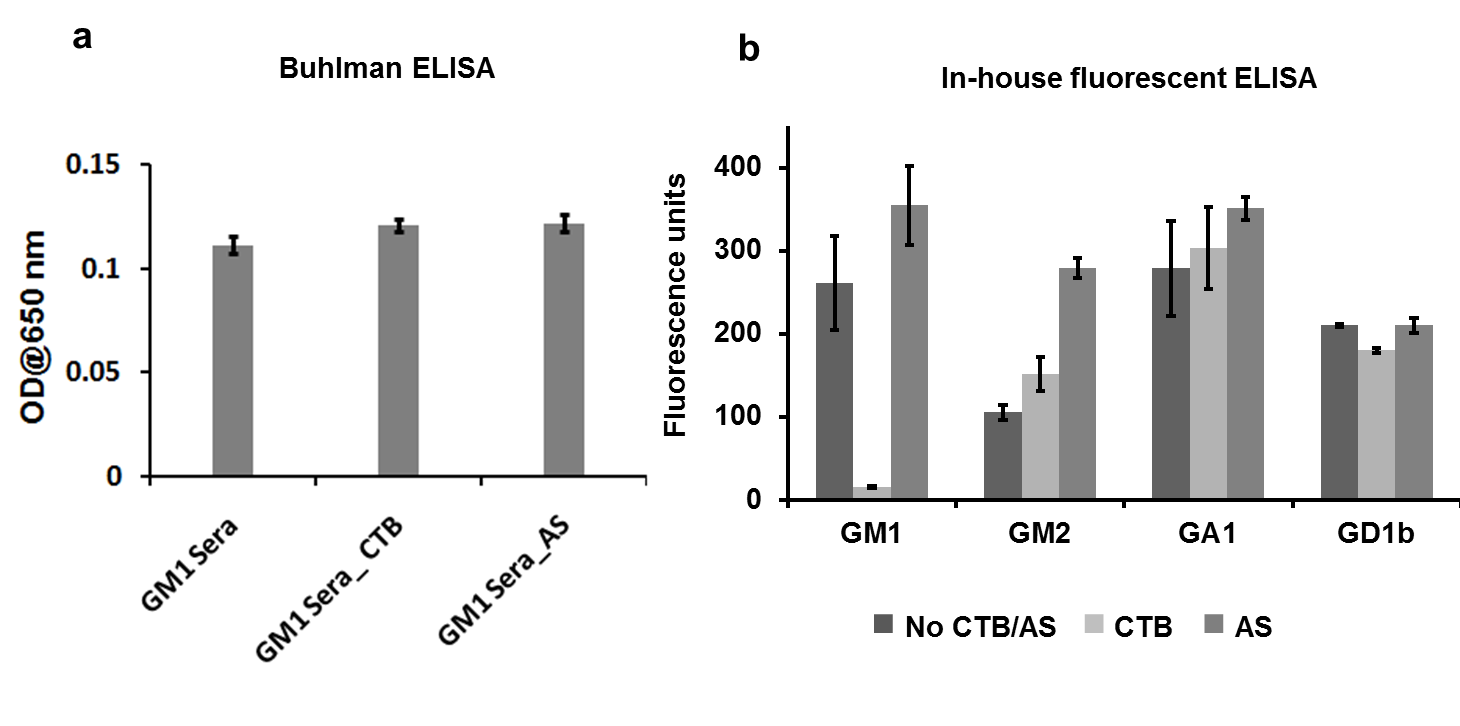

Supplement: Figure S2 — Testing epitope specificity using CTB and AS blockers in ELISA assays. GM1-specific sera standard was used as a sample in Buhlmann GM1 ELISA, and no blocking with either CTB or AS was observed (a). Effective blocking of GM1 by CTB was demonstrated in the in-house fluorescent ELISA, but no blocking effects of both CTB and AS were observed on the other gangliosides (b). Similar experiments using ganglioside Luminex bead array showed effective blocking of gangliosides with CTB and AS (Figure 2 in the main part). (TIF) [file pone.0042681.s002.tif]

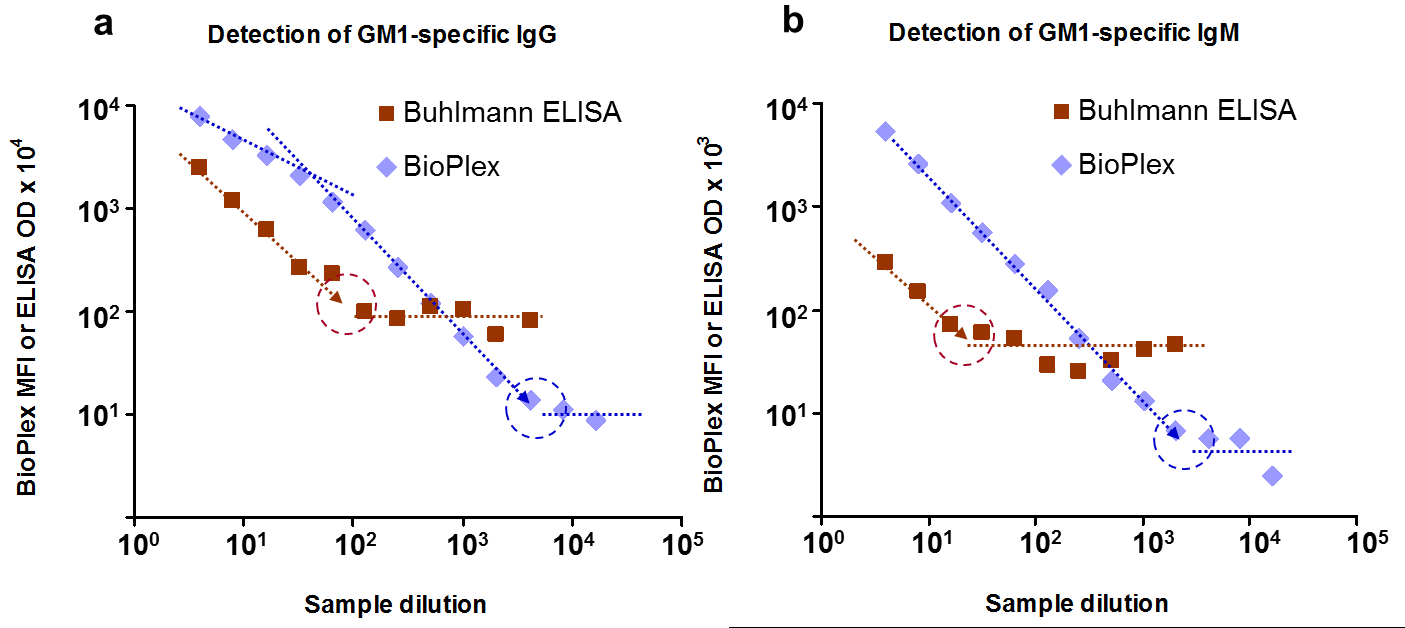

Supplement: Figure S3 — Concentration sensitivity to ganglioside-specific sera of BioPlex bead array, compared to Buhlmann ELISA. GM1-specific sera standard from Buhlmann kit was used as a sample in both BioPlex and ELISA experiments. Buhlmann GM1 ELISA was set using the kit protocol. In order to enable side-by side-comparison with Bioplex fluorescent data, ELISA optical density data were multiplied by arbitrary factors of 104 (IgG measurements, panel a) or 103 (IgM measurements, panel b). (TIF) [file pone.0042681.s003.tif]
